# Parallel Optimal Calibration of Mixed-Format Items for Achievement Tests

**DOI:** 10.1007/s11336-024-09968-3

**Published:** 2024-04-15

**Authors:** Frank Miller, Ellinor Fackle-Fornius

**Affiliations:** 1 Department of Statistics, Stockholm University, 10691 Stockholm, Sweden; 2 Department of Computer and Information Science, Linköping University, 58183 Linköping, Sweden

**Keywords:** Achievement tests, Calibration, Mixed-format items, Optimal design, Swedish national test

## Abstract

When large achievement tests are conducted regularly, items need to be calibrated before being used as operational items in a test. Methods have been developed to optimally assign pretest items to examinees based on their abilities. Most of these methods, however, are intended for situations where examinees arrive sequentially to be assigned to calibration items. In several calibration tests, examinees take the test simultaneously or in parallel. In this article, we develop an optimal calibration design tailored for such parallel test setups. Our objective is both to investigate the efficiency gain of the method as well as to demonstrate that this method can be implemented in real calibration scenarios. For the latter, we have employed this method to calibrate items for the Swedish national tests in Mathematics. In this case study, like in many real test situations, items are of mixed format and the optimal design method needs to handle that. The method we propose works for mixed-format tests and accounts for varying expected response times. Our investigations show that the proposed method considerably enhances calibration efficiency.

**Supplementary Information:**

The online version contains supplementary material available at 10.1007/s11336-024-09968-3.

Many achievement tests are conducted regularly, such as international large-scale tests like the Programme for International Student Assessment (PISA) or the Trends in International Mathematics and Science Study (TIMSS). Regularly conducted tests are also common on a national level, e.g., for knowledge assessments in school or qualification tests for university. It is crucial to pretest items before they can be used in an operational test. An important part of pretesting is to determine item characteristics, like the probability to correctly solve the item for examinees with different abilities. The process of pretesting to learn about the item characteristics is called item calibration.

To achieve high quality in the item calibration, a large number of examinees and considerable time and resources are required. It is therefore wise to investigate if calibration can be optimized in order to extract as much information as possible about the items given restricted resources. We propose a method that allocates pretest items to examinees based on their anticipated knowledge. In essence, we will match examinees to pretest items according to their (estimated) abilities. This can be seen as stratified sampling, using ability as the stratification variable.

Calibration can be conducted in various contexts. One approach is to incorporate pretest items into an operational test, which can be a computerized adaptive test. If knowledge about the examinees’ abilities from operational items’ results is available, one can utilize these and assign the pretest items based on the abilities in an optimal way. van der Linden and Ren (2015) and Ren et al. (2017) have proposed and investigated such an optimal calibration design for a test with dichotomous items. He and Chen (2020) conducted a comparative analysis of different calibration designs for dichotomous items. Zheng (2016) describes an optimal calibration design for polytomous items using the generalized partial credit model.

The calibration designs investigated by Zheng (2016), van der Linden and Ren (2015), Ren et al. (2017), and He and Chen (2020) optimize the allocation of the pretest item for an individual examinee based on the accumulated data from all pretest items and the examinee’s ability. These designs are applied sequentially, meaning that whenever an examinee is due to receive a pretest item, optimization is performed to determine the single item that would benefit the most from calibration for that particular examinee. Ren et al. (2017) compared approaches for handling cases where small groups (batches) of examinees receive pretest items simultaneously; this is achieved by sequentially going through the batch and applying sequential optimization. In certain real testing situations, pretest item parameters can be updated batch-wise for small groups of examinees and it makes sense to optimize the allocation of items for the subsequent small group of examinees. For instance, tests which include a pretest section and are offered many or most days of a year, are suitable for such a sequential optimization. Examples of tests that possess suitable settings for sequential optimization are the Graduate Record Examinations (GRE) test in the US and the Swedish driving license test, although they do not currently make use of this type of optimal calibration.

On the other hand, some calibration tests are instead conducted in parallel (simultaneously) for a large number of examinees. Instead of going through all examinees sequentially to determine optimally their pretest items, a design optimization performed for the entire calibration test at once offers more flexibility and allows for a superior design selection. Examples of tests in which many examinees take pretest items in parallel are the Scholastic Aptitude Test (SAT) in the US and the Swedish SAT. The US SAT is offered a few times a year and may include a calibration section. For the Swedish SAT 40,000 to 60,000 examinees undertake the operational test and its calibration part on a single day during each term. Our proposed methodology is especially suitable for scenarios where many examinees take pretest items in parallel.

While focus is often put on pretesting items having the same format and being analyzed with the same statistical model, mixed-format tests are important in some contexts and the rationale for their usage has been discussed (Lin, 2012). When items with different formats are used in the same calibration test, the analysis of the results should also allow for the use of different models. Mao et al. (2022) have investigated designs aimed at optimizing operational tests containing multidimensional mixed-format items. However, investigations of optimal calibration designs have so far predominantly concentrated on a single item format.

The objective of this article is to elaborate an optimized calibration design for a mixed-format test administered in parallel for all examinees. Since our method can be seen as stratified sampling and since the stratification variable ability is related to the outcome of interest, it is expected that the efficiency increases compared to randomly assigning items to examinees. However, it is not known whether the efficacy gain is large enough to justify the use of this optimized stratified sampling. Additionally, some optimized methods are challenging to apply in real situations and our aim is to develop a design which is also practical to use in a real-world calibration scenario. Consequently, the scientific questions in this research are: (1) Does the developed optimized design yield a sufficiently enhanced efficiency compared to randomly allocating items to examinees? (2) Is the developed design feasible to implement in a real calibration situation?

The outline of the article is as follows. In Sect. 1, we describe the case study focusing on the calibration of the Swedish national test in Mathematics. Section 2 defines the IRT models for the different item formats and gives the details of the proposed optimal design method for calibration. In Sect. 3, we provide results for three cases; a theoretical case, an idealized yet more realistic case, as well as our national test case study. The article concludes with a summarizing discussion.

## Case Study: Swedish National Tests

National tests are administered in Swedish schools during Grades 3, 6, 9, and once between Grades 10 and 12, covering subjects such as Swedish, English, and Mathematics. Throughout these grade levels, the pupils receive items of varying format, including short answer (correct/incorrect) items, multiple choice items, and graded response items. Currently, the tests are still performed as paper-and-pencil tests, but work is ongoing to computerize the tests in the future.

Due to the importance of the national test for the individual examinee, and the potential distraction an additional pretest item might pose, concurrent calibration alongside operational tests is avoided. Instead, voluntary teachers participate with their pupils in separate calibration tests some weeks after the operational tests have been evaluated. Typically, addition of pretest items to operational tests offers the advantage of ensuring consistent motivation among examinees (Zheng, 2014). However, in this situation, teachers have the opportunity to motivate their pupils by emphasizing that all tests contribute to the final grading. Therefore we judge the risk of random responders due to the test being percieved as low-stakes (Van Laar & Braeken, 2022) to be relatively low in this context. Consequently, it was decided to conduct the calibration at a separate occasion. It is important to note that the utilization of a stand-alone calibration test with voluntary participants, as seen in our case study, might not be advisable in other contexts. Employing a stand-alone calibration test is however not a requirement to implement the method discussed in this paper. In general, our method is applicable even when pretest items are integrated into an operational test.

As a specific case study, we are using the Swedish national test in Mathematics for Grade 6. Previously, various test versions with sets of pretest items were compiled and then randomly assigned to classes. Usually, each item was included in exactly one version, and each version could be sent to several classes. Since the calibration test for Mathematics items in Grade 6 was now computerized for the first time, it was feasible to assign distinct versions to individual examinees even within class.

## Optimal Design Method

### Models for Items and Examinee Population

We assume that *n* items are to be calibrated. The items can be of mixed format; some can be dichotomous being graded as 0 or 1 point, and others can be polytomous items graded as $0, 1, \dots , m_i$ points, where $m_i$ is the maximum number of points for item *i* $(i=1,\dots n)$. We assume that each item can be described by an item response theory (IRT) model. If item *i* is dichotomous, the model is described by the probability to correctly answer item *i*, $p_i(\theta ) = p_i(\theta , \varvec{\beta }_i)$. Here, $\theta \in \mathrm{I\!R}$ is the ability of an examinee and $\varvec{\beta }_i$ is the vector of item parameters which we want to estimate in this calibration. Examples of dichotomous IRT models are the two-parameter logistic (2PL) or the three-parameter logistic (3PL) model; the latter is described by

$$\begin{aligned} p_i(\theta )=c_i + (1-c_i)/[1+\exp \{-a_i (\theta -b_i)\}], \quad \varvec{\beta }_i=(a_i,b_i,c_i)^T, \end{aligned}$$

and setting $c_i=0$ yields the 2PL model. The parameter $a_i$ is called discrimination, $b_i$ difficulty, and $c_i$ the (pseudo-)guessing parameter of item *i*.

**Fig. 1:**
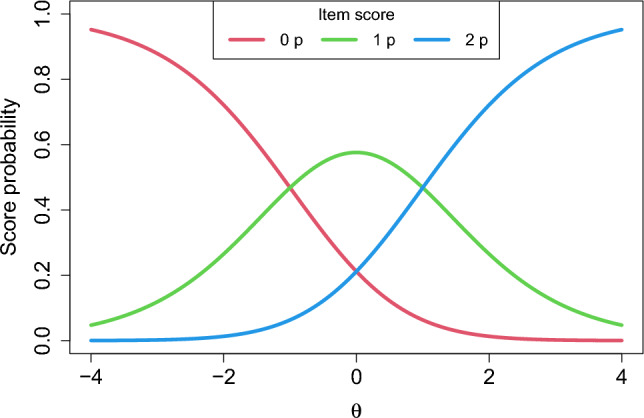
Response functions for an example GPCM for a 2-point item with $\varvec{\beta }_i=(a_i, b_{i1}, b_{i2}) = (1, -1, 1)$.

If item *i* is a polytomous, graded response item, the probability for an examinee with ability $\theta \in \mathrm{I\!R}$ to receive $k \in \{0, 1, \dots , m_i\}$ points for item *i* is $p_{ik}(\theta ) = P(Y_i = k|\theta , \varvec{\beta }_i)$, where $Y_i$ is the score on item *i*. An example of a polytomous IRT model is the generalized partial credit model (GPCM; Muraki, 1992; 1993), which can be described by

$$\begin{aligned} p_{ik}(\theta ) = \frac{\exp \left\{ \sum _{j=0}^{k} a_i \left( \theta - b_{ij}\right) \right\} }{ \sum _{g=0}^{m_{i}} \exp \left\{ \sum _{j=0}^{g} a_i \left( \theta - b_{ij}\right) \right\} }, \quad \varvec{\beta }_i=(a_i,b_{i1},\dots ,b_{im_i})^T, \end{aligned}$$

where $\sum _{j=0}^0 a_i\left( \theta -b_{ij}\right) =0$. For this formulation of the item category response function $p_{ik}(\theta )$ the probability of receiving score *k* is modeled as a function of the step difficulty parameters $b_{ij}$, interpreted as the difficulty in receiving score *k* given that one has reached the previous step $k-1$. The step difficulties are the points on $\theta$ where two consecutive item response functions intersect, see Fig. 1. There exist different formulations of the GPCM in the literature; see, e.g., Heinen (1993) and Nering and Ostini (2011) for alternative parameterizations and details about how this model relates to others.

In this paper, we assume that we have a population of voluntary examinees (in situations like our case study, usually there are, 1000–2000 examinees available). The population’s ability distribution can be described with a density $g(\theta )$; a reasonable assumption is often that *g* is the standard normal density.

### Test Versions

In real situations, the number of items (i.e., *n*) to be calibrated is typically large, such that only a smaller fraction of them can be allocated to a single examinee. Usually a certain number of test versions (i.e., *V*) are created, each containing a subset of the items. Each of the *n* items should be contained in at least one version, but could be included in several as well. A simple approach is to randomly divide the population into *V* subpopulations of approximately equal size and to assign each subpopulation to one test version. In contrast, the idea in this paper is that we assign the test versions to the examinees in an ability-dependent way. The first test version will be assigned to all examinees with ability smaller than some ability-bound $\theta _1$, the second to all examinees with ability from $\theta _1$ to below $\theta _2$ and so on. Hence, all examinees with ability $\theta \in [\theta _{v-1}, \theta _v)$ will be assigned to version *v*, $v=1,\dots ,V$ with $\theta _0=-\infty , \theta _V=\infty$. This means that versions with lower number will be assigned to examinees with lower ability. We assume here that the number of versions *V* is fixed (too large of a number might not be feasible) and that the ability boundaries $\theta _v, v=1, \dots , V-1$ are chosen in advance. Nevertheless, we will also investigate the impact of the choice of *V* in Sect. 3.2 and 3.3.

### The Calibration Design

The design of our calibration involves describing which items are included in which version. Formally, an $n\times V$-matrix $(d_{iv})$ of 0’s and 1’s defines the design:

$$\begin{aligned} d_{iv}= \left\{ \begin{array}{ll} 1, &{} \text{ if } \text{ item } i \text{ is } \text{ used } \text{ in } \text{ version } v, \\ 0, &{} \text{ otherwise. } \end{array} \right. \end{aligned}$$

The versions have to fulfill certain restrictions, e.g. such that the test length is adequate.

#### Fixed Length Versions

One reasonable design restriction is to limit the number of items in each version by *d* items:

1 $$\begin{aligned} \sum _{i=1}^n d_{iv} \le d \text{ for } \text{ all } v=1,\dots ,V. \end{aligned}$$

#### Versions with Target Time

While the above restriction might be meaningful in cases where all items are of similar structure, it is often desired to calibrate items which require different response times until solved (Ali & Chang, 2014; He et al., 2021). Therefore, we introduce $t_i$ as the expected response time for item *i*, given in some time unit. A design restriction is then

2 $$\begin{aligned} \sum _{i=1}^n d_{iv} t_i \le T, \end{aligned}$$

where *T* is the target time for the test. Note that the case (1) with a fixed length version is a special case of this case if we set $t_1=\dots =t_n=1$ and $T=d$.

In real situations, there could be further design restrictions, e.g., that the number of items with a specific content is restricted. While these restrictions can easily be incorporated, we do not extend the notation here for the sake of simplicity. Also, our case study which we will come back to in Sect. 3.3 only had restrictions of type (2).

### Information

In the context of calibration tests, we have not unrestricted access to voluntary examinees with desired ability levels. Therefore, application of traditional optimal design methodology is not possible, as pointed out, e.g., by Zheng (2014) and van der Linden and Ren (2015). Due to this, we use optimal design theory based on finite population sampling (Wynn, 1982) as described by Ul Hassan and Miller (2019). The available finite population of voluntary test takers is described by a probability density $g(\theta )$. Let $h_i(\theta )$ be the sub-density describing the volunteers allocated to item *i*. With the notation introduced before, the sub-density is

$$\begin{aligned} h_i(\theta )=\sum _{v=1}^V d_{iv} \varvec{1}\{\theta _{v-1} \le \theta < \theta _v\} g(\theta ), \end{aligned}$$

where $\varvec{1}\{\dots \}$ is the indicator function being 1 if the condition in brackets is fulfilled and 0 otherwise.

The information matrix for $\varvec{\beta }_i$ for a dichotomous item *i* is then

3 $$\begin{aligned} M_i = \int p_i(\theta )\{1 - p_i(\theta )\}~ \left( \frac{{\partial \eta _i (\theta )}}{{\partial \varvec{\beta _i }}}\right) {\left( \frac{{\partial \eta _i (\theta )}}{{\partial \varvec{\beta _i}}}\right) ^T}~ h_i(\theta ) ~ {\textrm{d}}\theta , \end{aligned}$$

where $\eta _i (\theta ) = \log \left[ {p_i(\theta )}/\{1 - p_i(\theta )\}\right] ,$ see Ul Hassan and Miller (2019). The information matrix for the 2PL and 3PL model has been elaborated before; see, e.g., Ul Hassan and Miller (2019) and Ul Hassan and Miller (2021), respectively.

For polytomous items, the structure of the information matrix under various models is given in Holman and Berger (2001) and for the nominal response model in Berger et al. (2000). In general, with examinees distributed according to $h_i(\theta )$, it has the following format

4 $$\begin{aligned} M_i = \int \left( \frac{{\partial l_i(\theta )}}{{\partial \varvec{\beta }_i }}\right) {\left( \frac{{\partial l_i(\theta )}}{{\partial \varvec{\beta }_i }}\right) ^T} ~ h_i(\theta ) ~ {\textrm{d}}\theta , \end{aligned}$$

where $l_i(\theta )=\sum _{k=0}^{m_i} \log p_{ik}(\theta )$. An example of the $3 \times 3$ GPCM information matrix for a three categories item with the element details worked out, is given in Appendix A1.

We are interested in estimating not just one, but all of the item parameter-vectors $\varvec{\beta }_i$ with good precision. This means that the parameter-vector of interest is $\varvec{\beta }=(\varvec{\beta }_1^T, \dots , \varvec{\beta }_n^T)^T$. We assume local independence of the items and therefore, the total information matrix for $\varvec{\beta }$ is block-diagonal: $M = \textrm{diag}\left( M_1, \dots , M_n\right) .$

We highlight that the information in this article is the information about item parameters, only. We do not include information about ability parameters, which is necessary, for example, in computerized adaptive tests where items are selected with the purpose to maximize information about the examinees’ abilities. We consider only a calibration test or only the pretest items within a larger test. We estimate the item parameters of pretest items while assuming that ability estimates are based on operational items, only.

### Design Optimization

The desire in optimal design is to maximize the information. Since we have an information matrix, we need to choose an optimization criterion (Atkinsson et al., 2007). We have chosen here D-optimality, which optimizes the determinant of the information matrix and has several good properties (Atkinsson et al., 2007). For this criterion, we have

$$\begin{aligned} \det (M) = \prod _{i=1}^n \det (M_i). \end{aligned}$$

If only very few examinees would calibrate one of the items, one factor in the product would be very small, thus making the product small. Therefore, the product structure ensures that sufficient emphasis is placed on each item.

The IRT models considered here are nonlinear models. A typical issue with optimal designs for such models is that the information matrix depends on the parameters $\varvec{\beta }$ to be estimated. One way of dealing with this is to determine the optimal design when setting the unknown parameter in the information matrix to an anticipated (best guess) value $\varvec{\beta }^{(0)}$. This approach is called local optimality. For our case study, this approach was reasonable since anticipated values for the parameters exist, see further the discussion in Sect. 3.3.

To compute optimal designs numerically, we maximize $\det (M)$ over all $n\times V$-0-1-matrices which fulfill the required restrictions. Combinatorial optimization algorithms (see, e.g., Givens & Hoeting, 2012, Chapter 3) can be used for that. For the results shown in Sects. 3.2 and 3.3, we applied a simulated annealing algorithm.

### Random Design and Efficiency

The quality of the derived optimal designs will be compared against that of a random design, which allocates the items randomly to the examinees, independent of their abilities. Formally, the random design is characterized by a density $h^R$ which is a constant fraction of the population density *g* for each item. In case of a fixed length design, each item has probability *d*/*n* to be allocated and $h^R(\theta ) = g(\theta ) \cdot d/n$. If versions with target time *T* are used, each item has probability $T/\sum _{i=1}^n t_i$ to be allocated and $h^R(\theta ) = g(\theta ) \cdot T/\sum _{i=1}^n t_i$.

The relative D-efficiency of a design with information matrix *M* versus the random design with information matrix $M^R$ is defined as

5 $$\begin{aligned} {\textrm{RE}}_D = \left( \frac{\det (M)}{\det (M^R)} \right) ^{1/p} \end{aligned}$$

where *p* is the total number of parameters for all items (Berger and Wong, 2009). If ${\textrm{RE}}_D>1$, the random design needs $({\textrm{RE}}_D-1)*100$ percent more examinees than the compared design to obtain estimates with similar precision. We will also compute relative D-efficiencies ${\textrm{RE}}_{D,i}=\left( {\det (M_i)}/{\det (M_i^R)} \right) ^{1/p_i}$ for an individual item *i*. Note that ${\textrm{RE}}_D=\left( \prod _{i=1}^n {\textrm{RE}}_{D,i}^{p_i}\right) ^{1/p}$; ${\textrm{RE}}_D$ is equal to a weighted geometric mean of the $RE_{D,i}$, weighted with the number of parameters in each sub-model.

Note that we only consider designs for optimisation where the items are administered in *V* versions and that we do not allow full flexibility. In contrast, we do not have this requirement for the random design and it might not be possible to create *V* versions of the random design which fulfill the restrictions and ensure that each item has the same probability of selection. Therefore, the efficiency of the optimal design compared with the random design for *V* versions can be worse in some cases, $RE_D<1$, especially if *V* is small.

### Unrestricted Design Optimization

In order to explain the results which we will obtain for our described optimization method, we will first consider the case of traditional, unrestricted design optimization. For this, we pretend that we have access to an arbitrary number of examinees with every possible ability. A design is then the intention to calibrate item *i* with examinees having abilities $\theta _{i1}, \dots , \theta _{i,n_i}$ for $n_i$ ability-levels (called “design points”). The proportion of examinees at $\theta _{ij}$ is $w_{ij}$ with $w_{ij} \ge 0$ and $\sum _{j=1}^{n_i} w_{ij}=1$. The design can then be summarized as

$$\begin{aligned} \xi _i = \left( \begin{array}{ccc} \theta _{i1} &{} \dots &{} \theta _{i,n_i} \\ w_{i1} &{} \dots &{} w_{i,n_i} \end{array}\right) . \end{aligned}$$

Instead of (3), the information matrix for $\varvec{\beta }_i$ for a dichotomous item *i* is then:

$$\begin{aligned} M_i = M_i(\xi _i) = \sum _{j=1}^{n_i} p_i(\theta _{ij})\{1 - p_i(\theta _{ij})\}~ \left( \frac{{\partial \eta _i (\theta _{ij})}}{{\partial \beta _i }}\right) {\left( \frac{{\partial \eta _i (\theta _{ij})}}{{\partial \beta _i }}\right) ^T}~ w_{ij}. \end{aligned}$$

Similarly, the information matrix for a polytomous item becomes a sum instead of (4). The D-optimal design determines $n_i, \theta _{i1}, \dots , \theta _{i,n_i}, w_{i1}, \dots w_{i,n_i}$ such that $\det (M)=\det \{\textrm{diag}\left( M_1,\dots ,M_n\right) \}=\prod _{i=1}^n \det (M_i)$ is maximized.

## Results

In Sect. 3.1, we start by showing the unrestricted D-optimal designs for the 2PL, 3PL, and GPCM models for three categories. Next, we take the restriction by the available population into account in Sect. 3.2. Here, we assume idealized situations with one (or two) item-type(s) and uniformly spread item difficulties. The results show a meaningful structure corresponding to the results for unrestricted optimal designs. In Sect. 3.3 we demonstrate that our approach can also handle complicated mixed-type situations. We apply the design optimization for the mixed-format pretest items in the Swedish national test in Mathematics. This illustrates how the results from the idealized situations generalize to a realistic case.

### Theoretical Results for the Unrestricted Design Case

As mentioned previously, the optimal designs for nonlinear models depend on the parameters and we adopt locally optimal designs here. Nevertheless, when we consider unrestricted design optimization, there is no need to investigate all combinations of parameter values. We can without loss of generality fix (one of) the difficulty parameter(s). For other values of this parameter, the D-optimal designs are shifted. We can therefore restrict us to $b_i=0$ in the 2PL- and 3PL-model and to $b_{i1}+b_{i2}=0$ in the GPCM for three categories. Further, we can also just consider the discrimination parameter as $a_i=1$ in all models (models with other parameter values can then be obtained by scaling). This shifting and scaling is possible due to the invariance of the D-optimality criterion, see Idais and Schwabe (2021) for more background.

#### 2PL-Model

For estimating the discrimination (slope), it is intuitively reasonable that information should be collected from examinees which have abilities somewhat below and somewhat above the difficulty of the item. Indeed, the D-optimal design is to include examinees with ability $\theta _1=b_i-1.543/a_i$ and with ability $\theta _2=b_i+1.543/a_i$ (two design points) and to include equally many for each ability. This design is well known, see Abdelbasit and Plackett (1983).

#### 3PL-Model

Stocking (1990) investigates which examinees are most informative for estimating each of the three parameters in the 3PL model; see, e.g., her discussion on page 474. Low ability examinees are needed for estimating the guessing parameter well and again examinees somewhat below and above the difficulty are needed for estimating the difficulty and the discrimination. For the D-optimal design, it can be shown that the locally D-optimal design has one design point in $\theta =-\infty$ which has the purpose to estimate the guessing parameter. For each guessing parameter, the optimal design has three design points and the other two depend on the guessing parameter value. Since a three point design is minimally supported (with less than three points, the parameters are not estimable), the weights in the design points have to be equal, i.e., they are 1/3, see Silvey (1980).

#### GPCM with Three Categories

Here, we present results for unrestricted D-optimal designs for the GPCM with three categories. These illustrate how the number of design points depend on the parameters. To the best of our knowledge, these results have not been reported in the literature before.

Due to symmetry reasons, the D-optimal design is symmetrical around $(b_{i1}+b_{i2})/2$. Numerical results for optimal design points depending on $b_{i2}-b_{i1}$ are shown in Appendix A2. These results show that a two-point design is optimal for $a_i(b_{i2}-b_{i1}) \le 1.51$, a three-point design is optimal for $1.51 < a_i(b_{i2}-b_{i1}) \le 5.25$ and a four-point design for larger values. In the case of an optimal two-point design, it is minimally supported, and both points have equal weight 0.5.

### Results for Idealized Situations with a Single Type of Items and a Mixed-Format Test

To investigate the properties of the optimal design, we first consider a set of simplified settings starting with situations with a single type of item. We assume for illustration that $n=60$ items have to be calibrated and $V=40$ test versions can be created. Each version should be allocated to 2.5% of the examinees. Assuming standard normal distributed abilities, the ability limits are quantiles of the standard normal distribution:

6 $$\begin{aligned} \theta _1=z_{0.025},\, \theta _2=z_{0.05},\, \dots , \, \theta _{39}=z_{0.975}. \end{aligned}$$

A fixed length restriction (1) is required allowing for $d=9$ items per version.

For the items, we consider three models:

- All items follow a 2PL model and we want to estimate these two parameters. The anticipated item parameters are in this situation: Difficulty parameters equidistantly between – 2 (Item 1) and 2 (Item 60); the discrimination parameter is 1 for all items. These anticipations are used to calculate the optimal design, but the true item parameters are still unknown and need to be estimated.
- All items follow a 3PL model with anticipated difficulty and discrimination parameters as before and an anticipated guessing parameter of 0.2 for all items.
- All items follow a GPCM for categories 0, 1, and 2. The anticipated discrimination parameter is 1 for all items; the anticipated parameter $b_{i1}$ is equidistantly between $-$1.5 (Item 1) and 2 (Item 60) and $b_{i2}=b_{i1}-0.5$ for each item.

Note that the anticipated difficulty parameter(s) are increasing from Item 1 to Item 60 in all three situations. Therefore, our anticipation which we use for computation of the optimal design is that the items are sorted by difficulty.

**Fig. 2:**
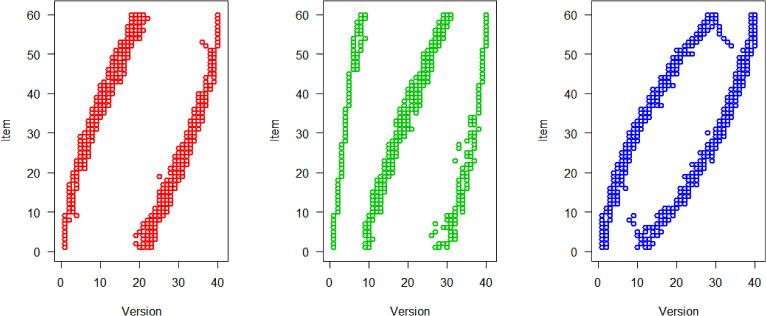
Optimal design for 2PL model (left, red), 3PL model (middle, green), and GPCM (right, blue), 40 versions, and 9 items per version.

Figure 2 shows the computed optimal designs for the three situations respectively. As mentioned before, a design is represented by an $n\times V$-matrix of 0’s and 1’s. We show the optimal design as figure where a dot is shown if and only if the item was used in the version. We see for all models that the examinees with low ability (low number versions) receive easier items (low number items) and high ability examinees receive more difficult items.

Fixing an arbitrary item in the 2PL model (left panel in Fig. 2), we see that usually two groups of examinees are chosen: a lower ability and a higher ability group. E.g., Item 1 is allocated to Version 1 (examinees with $\theta < z_{0.025}=-1.96$) and to Version 20-23 (examinees with $z_{0.475}=-0.06 \le \theta < z_{0.575}=0.19$). That two groups are needed corresponds to our theoretical result for the unrestricted case (Sect. 3.1). Now, fixing an item in the 3PL model (middle panel in Fig. 2), we see that three groups of examinees are chosen for most items in accordance with the theoretical results for the unrestricted case. Finally, fixing an item in the GPCM (right panel in Fig. 2), two groups of examinees are usually chosen. Also this is in accordance with the theory in Sect. 3.2, since $a_i(b_{i2}-b_{i1})=-0.5<1.51$ in our model. In Appendix A3, we present two additional scenarios using parameter sets that yield three or four groups of examinees. Note, however, that these parameter sets are more theoretical in nature.

**Fig. 3:**
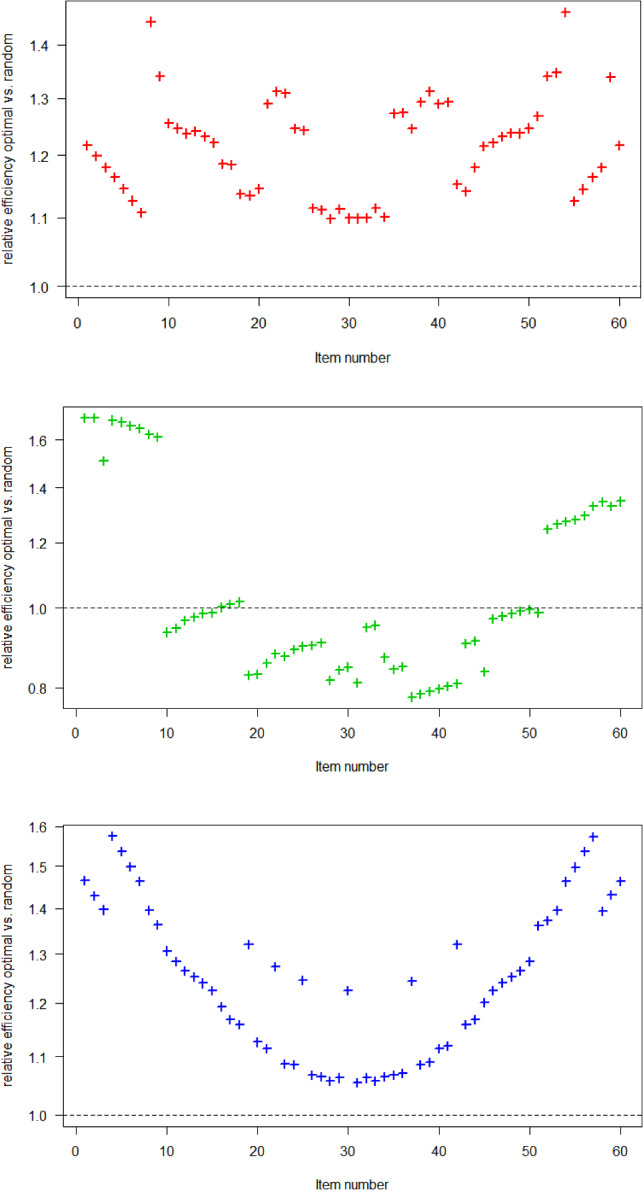
Relative efficiencies for 2PL (first panel), 3PL (middle panel), and GPCM (last panel) model, 40 versions, and 9 items per version. Optimal versus random design.

We now compare the information obtained by the optimal design and a random design. Figure 3 shows the relative efficiency, the ratio of the criterion values $\det (M_i)/\det (M_i^R)$, for each of the 60 items (easy left, difficult right) in each of the three models. For the 3PL and GPCM, we see that the optimal design especially improves the precision of the easy and difficult items. The precision gain is not as significant for items with moderate difficulty; for the 3PL model, there is even a precision loss for items with moderate difficulty. For the 2PL model, we notice a general improvement in precision across all items. However, in this case, the distinction between easy and difficult items is not as prominent as observed in other models. When examining similar plots for scenarios involving 20 or 60 versions, a consistent trend emerges: easy and difficult items show greater efficacy gains from the optimal design. Detailed figures for these cases are provided in the supplementary material S1.

In Appendix A4, we show the relative efficiencies for the item parameters across all items. While the GPCM shows an efficiency gain for all item parameters, both efficiency loss and gain are observed for the 2PL and 3PL models. It is important to note that the optimal design was computed to maximize the determinant of the information matrix. If our focus is specifically on the performance of two or three item parameters, a more tailored criterion like A-optimality might be preferred.

In this article, our emphasis lies on the information matrix and the efficiencies derived from it. The information matrix provides asymptotic information concerning standard errors, particularly in the context of large sample sizes. Asymptotically, biases are eliminated. However, for finite sample sizes, biases can affect estimates for the models under consideration. Therefore, we conducted a simulation study for the 2PL and 3PL models to explore biases for all item parameters, see supplementary material S3. We see some bias in all item parameter estimates, yet the D-optimal design demonstrates its ability to reduce the bias observed with a random design. The optimal design’s selection of examinees, aimed at minimizing the asymptotic variance of estimates, ensures the acquisition of sufficient information about the parameters, thereby avoiding extreme estimates or non-convergence issues.

The relative D-efficiency based on all 60 items via Formula (5) for the 2PL, 3PL, and GPCM are 1.22, 1.04, and 1.25, respectively. In Table 1, we show how the averaged relative efficiency depends on the number of versions. A larger number of versions gives more flexibility and can therefore increase the relative efficiency. On the other hand, the development of too many versions might pose challenges in development and administration. We see here that the relative efficiency is not much affected by *V* for the 2PL and GPCM. In contrast, it is highly depending on *V* for the 3PL model. For $V\le 30$, the random design is even better than the optimal design since the random design is not applied version-wise.

**Table 1:** Relative efficiency of optimal design versus random design depending on model (2PL, 3PL, or GPCM) and number of versions ($V=12, 15, 20, 30, 40, 60, 100$), averaged over all 60 items.

	$$V=$$						
Model	12	15	20	30	40	60	100
2PL	0.86	1.14	1.20	1.22	1.22	1.22	1.22
3PL	0.27	0.47	0.77	0.96	1.04	1.11	1.15
GPCM	1.11	1.22	1.23	1.24	1.25	1.25	1.25

**Fig. 4:**
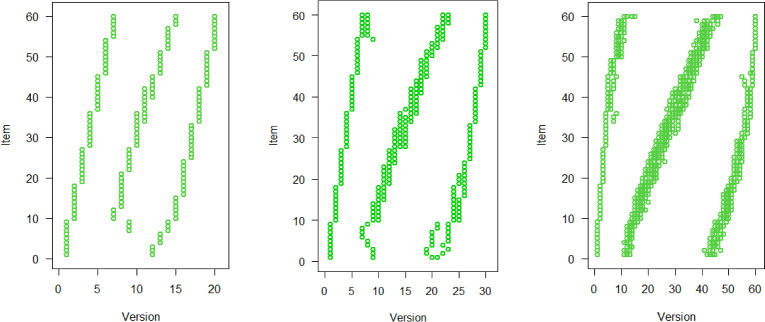
Optimal design for the 3PL model and $V=20, 30, 60$ versions (left, middle, right, respectively), and 9 items per version.

To comprehend why the performance of the optimal design for the 3PL model relies heavily on *V*, we illustrate the optimal designs for $V=20, 30, 60$ in Fig. 4. A larger *V* implies higher flexibility; hence, we consider $V=60$ as our benchmark. When *V* is smaller, our aim is to allocate similar groups of examinees to the items, though achieving this comprehensively is not fully possible. Let us take Item 29 as an example: The optimal design for $V=60$ suggests assigning this item to versions 4, 23 to 27, and 53 to 55. This allocation entails including a group of low-ability examinees (1/60 of the total population), a middle-ability group (5/60), and a high-ability group (3/60). Upon reducing the number of versions to $V=20$, we still require three distinct groups of examinees with varying abilities to maintain reasonable efficiency. However, we can usually have only three versions per item. Therefore, we must oversample the low-ability group (1/20 instead of 1/60) and undersample the middle-ability group (1/20 instead of 5/60). Since we have to approximate the $V=60$-design with a considerably different $V=20$-design, there is a high impact on efficiency.

**Fig. 5:**
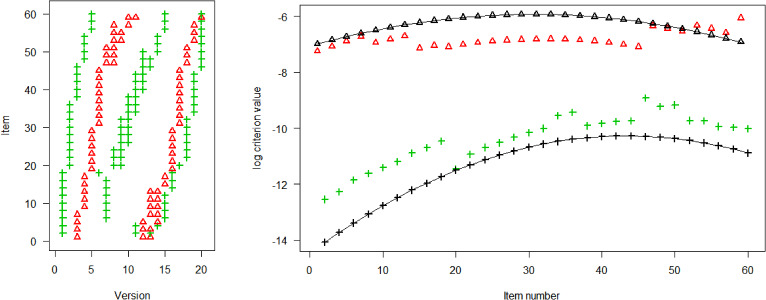
Optimal design (left panel) and item information (right panel). Red $\Delta$ = 2PL items, green $+$ = 3PL items. Right panel: colored without joining lines for optimal design, black with joining lines for random design. Mixed-format test with 2PL and 3PL items. 20 versions and 9 items per version.

Finally, we consider a test of mixed format consisting of each 30 2PL- and 3PL-items. The discrimination parameter is 1 and the guessing parameter 0.2 for the 3PL-items. Difficulty is equidistant between -2 and 2 for each model. We consider the case of 20 versions which appeared to be challenging for the 3PL-model. It would be misleading to assume that in this mixed-format test, only the 2PL-items and not the 3PL-items will benefit from the optimal design. For the random design, the 2PL-items have higher information (higher $\det (M_i)$) compared to the 3PL-items. In order to optimize the overall information $\det (M)$, the optimal design puts more effort on the 3PL-items leading to increased efficiencies for those items while the efficiencies of the 2PL-items are similar for both designs, see Fig. 5. The dependency of relative efficiencies on the number of versions used is shown in Table 2.

**Table 2:** Relative efficiency of optimal design versus random design for mixed 2PL and 3PL model depending on number of versions ($V=12, 15, 20, 30, 40, 60, 100$), averaged over all 60 items (first row) or all 30 2PL or 30 3PL items in second and third row.

	$$V=$$						
Item type	12	15	20	30	40	60	100
All items (2 &3PL)	0.54	0.93	1.08	1.18	1.21	1.24	1.26
2PL-items	0.37	0.72	0.78	0.93	0.96	1.02	1.03
3PL-items	0.70	1.09	1.34	1.38	1.42	1.41	1.43

### Results for the Calibration for Swedish National Test in Mathematics

A set of 85 items was selected for calibration in May and June 2022 where voluntary teachers and their classes with a total of around 1600 pupils, agreed to participate in a calibration test. All pupils had participated in their ordinary national test in Mathematics and the teachers reported their total points from the national tests. The 85 items were a subset of items pretested in a calibration test in Spring 2021. The results from the pretest had been analyzed with IRT analysis and consequently estimates of item parameters were available for all items. The estimated parameter values are shown in Table 5 in Appendix 5. Each of these pretest items was pretested by on average 229 pupils (range from 189 to 259).

The 85 items were of mixed format such that both 2PL-models (for short answer, correct/incorrect-items), 3PL-models (for multiple choice items), and GPCM (for items where 2 points could be achieved) were used. Some of the items consisted of Part a and b or Part a, b, and c of a question and had to be included together in the same version. We refer to these as item groups. The models used for the 63 item groups are summarized in Table 3; for details, we refer to Appendix-table 5.

**Table 3:** Item types in the calibration test for Swedish national test in Mathematics.

Item group type	Model used	Number
Single, short answer (correct/incorrect)	2PL	22
Single, multiple choice	3PL	9
Pair, short answer (correct/incorrect)	2PL+2PL	10
Triple, short answer (correct/incorrect)	2PL+2PL+2PL	6
Single, graded response (0, 1, or 2 points)	GPCM	16
Sum		63

Since it was anticipated that different item groups will have different response time, the test developers estimated the average response time for each item group, using also the experience from the pretesting. These times $t_i, i=1, \dots 63,$ were between 2 and 8 min. The target time for a whole calibration test was set to $T=40$ minutes, and design restriction (2) was used. It has been highlighted, e.g., by He et al. (2021) that it is important to take the expected response time into account when optimizing item calibration. He et al. (2021) explicitly define a D-optimality criterion per time unit in their situation of continuous online calibration which they call DT-optimality. In our situation of parallel testing of pretest items only, we can optimize using D-optimality and achieve information by time since the optimization tries to collect as much information as possible given a time constraint *T* for the test versions.

**Fig. 6:**
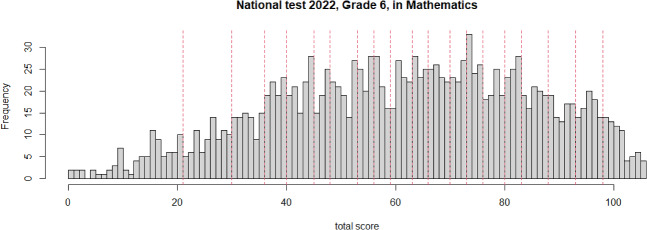
Swedish national test in Mathematics: Results from Grade 6 in 2022 of the pupils who participated in the calibration test; red vertical lines: division into groups for the $V=20$ calibration versions.

Figure 6 shows a histogram of the total results (raw scores) in the ordinary national test of the around 1600 pupils who participated in the calibration test. In order to transform these raw scores to an ability scale, we normalized them based on the percentiles such that the normalized scores follow a standard normal distribution; see, e.g., Kolen and Brennan (2014), Section 9.5.2.; according to Petersen et al. (1989), this is an often-used transformation. It was decided to create $V=20$ versions with 5% of the pupils allocated to each version, i.e. using the limits $\theta _1=z_{0.05},\, \theta _2=z_{0.1},\, \dots , \, \theta _{19}=z_{0.95}$ between the versions. Transferring these limits back to the raw scores, the red vertical dashed lines in Fig. 6 indicate the limits between the 20 versions.

**Fig. 7:**
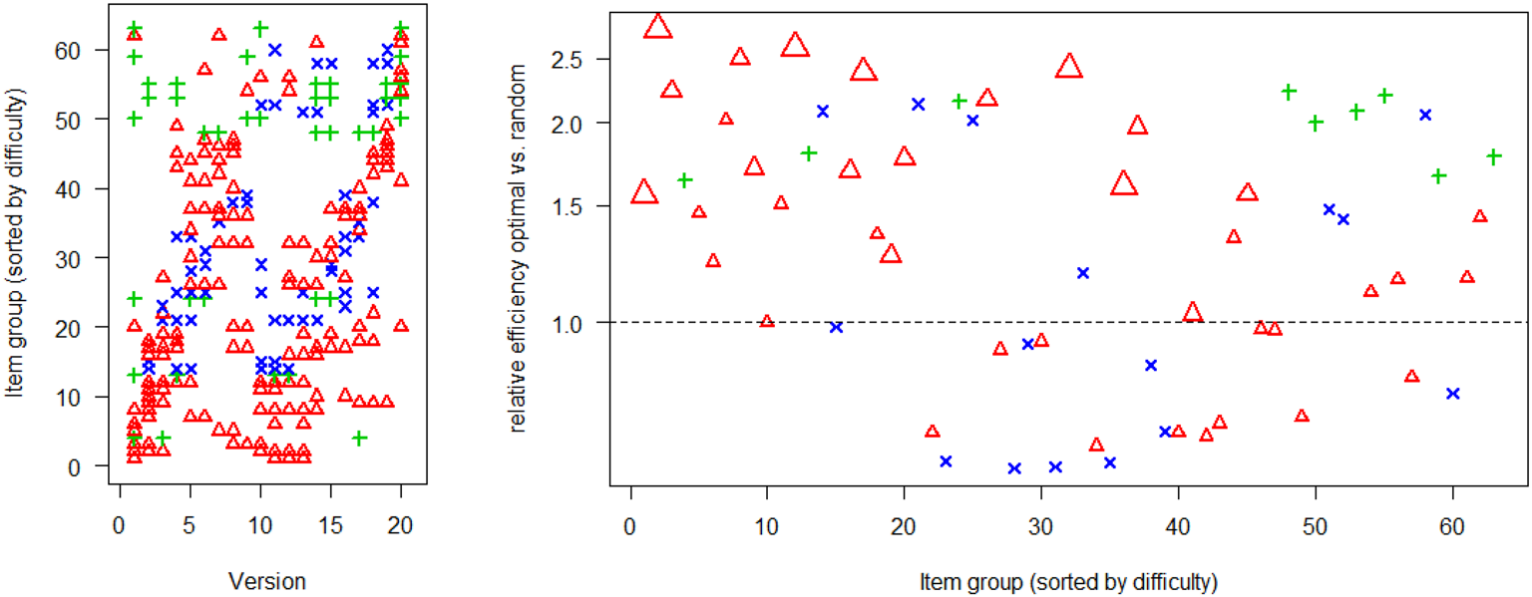
Calibration test for Swedish national test in Mathematics: Optimal design (left) and relative item efficiencies (right). Symbols and colors: red $\Delta$ for 2PL item groups, green $+$ for 3PL items, blue $\times$ for GPCM items. Size of symbols in right panel: small $\Delta$ = single 2PL item, medium $\Delta$ = group of two 2PL items; large $\Delta$ = group of three 2PL items.

Figure 7 shows the optimized design and item efficiencies. The 63 item groups were sorted by difficulty. For this figure, we defined the difficulty of the different item (group) types via the *b*-parameters in the following way: Difficulty for a group of 2PL-items was defined as average of the $b_i$-parameters, difficulty of a 3PL-item was defined as $b_i$, which is the ability where the probability of a correct result is $(1+c_i)/2$, and difficulty of a GPCM-item was defined as average of the two $b_{ig}$-parameters, i.e. the ability which has an expected result of one out of two points. When we focus on 2PL- and GPCM-items, we see roughly again (as in Sect. 3.2) two ability intervals of pupils allocated to many of those items, and that both intervals shift to higher abilities with more difficult item groups. Note that the estimated item parameters for the GPCM-items have $b_{2i}-b_{1i}<0$ except of one item which has $a_i(b_{i2}- b_{i1})=0.65<1.51$ and from a theoretical perspective, we expect two ability intervals allocated to the items. When we focus on the 3PL-items, we see that most of them are included in Version 1 or 2 for efficient estimation of the guessing parameter. The relative efficiencies of the items (optimal versus random design) are larger than 1 for the majority of the items and they tend to be higher for the easier items. The reason why we do not see increased relative efficiency for the most difficult item is that there were not so many very difficult items included in this calibration. The comparison of the item-efficiencies is here more complicated since different items have different expected response times. It is possible to place more 2-minute items in a version compared to items with longer response time. More information can be collected if more of the shorter items are used. Therefore, the shorter items are used in more versions than the longer items (see the supplementary material S2). Since the GPCM items tend to require longer time, some of them are used in only a few versions and their efficiency is lower compared to other items (see also the supplementary material S2).

Averaging over the relative efficiencies using the geometric mean gave an averaged relative efficiency of optimal design versus random design of 1.44. This means that roughly 44% more pupils would be necessary if the random design would have been used instead of the optimal design, or roughly 2300 instead of 1600 pupils.

**Table 4:** Calibration test for Swedish national test in Mathematics: Relative efficiency of optimal design versus random design ($V=12, 15, 20, 30, 40, 60, 100$), averaged over all items (first row) or all 2PL, all 3PL, and all GPCM items in other rows.

	$$V=$$						
Item type	12	15	20	30	40	60	100
All items	1.31	1.40	1.44	1.47	1.47	1.48	1.48
2PL-items	1.29	1.46	1.52	1.55	1.56	1.59	1.58
3PL-items	1.94	1.90	1.93	1.92	1.86	1.85	1.87
GPCM-items	1.10	1.08	1.06	1.10	1.12	1.09	1.10

## Discussion

Both our case study and investigations of various idealized situations demonstrated that the total efficiency of parameter estimates can be substantially improved when an optimal calibration design is used. This optimal design can be applied in a parallel, non-sequential setting. To be able to use this type of optimal design it is necessary that information about the examinees’ ability is available. This ability information can be sourced from prior test results stored within a database. Alternatively, it can originate from preceding items within a test, where pretest items are integrated with operational ones. Also, preliminary information about item parameters is required, derived either from previous calibrations (like in our case study) or through expert judgements. If no information is available about the examinees’ abilities or the item parameters, the methods discussed herein cannot be used and random allocation of items to examinees should be done instead.

When calibration tests are repeatedly conducted, each administered in parallel for a larger number of examinees with known ability information, our method can be implemented as follows: In the initial calibration test conducted without prior item parameter information, items are randomly allocated. After this test, the results undergo analysis; some items are discontinued due to unfavorable properties, while others are directly chosen for inclusion in the item bank for future use as operational items. Certain items are selected for further pretesting in subsequent calibration tests; the parameter estimates of these items can be used to compute the optimal design for the subsequent calibration study. New items introduced for the second calibration test are randomly distributed among examinees. After the second calibration test, an analysis is done and item parameters are updated again for those items to be used in the third calibration. The updated parameters are used for a new optimal design calculation for the third calibration test, and we continue with this process. It is important to note that recalculating the optimal design for each new test is necessary for two reasons: Firstly, the anticipated item parameters change from one test to the next. Additionally, even if these parameters would not change, the optimal design for an individual item depends on the other items which belong to the calibration pool.

Through our case study on the Swedish national test in Mathematics, we demonstrated that it is possible to implement the design in a real calibration setting. Practical requirements like different expected response times and the necessity to keep specific items together in a group could be implemented as well. Creating a number of versions made it possible to check if a specific combination of items would be strange. If this would have happened, another optimization avoiding such combinations could have been run. A difference compared to the usual setting, where versions are randomly distributed to classes, the teachers had to report the results of the pupils from their national tests in advance. This was an extra burden, but was considered acceptable. In future, it might be possible to think about ways to collect this information in a more automated manner. When the test administration is fully digitized there may be technical solutions available.

Using a random design is especially negative for very easy and very difficult items. The optimal design can clearly improve the quality of the estimates for these items. This was true for all models considered in this research.

In this article, we used locally optimal designs, which require an initial guess of the parameter values and are optimal if the true parameter values are equal to the guessed values. An alternative to locally optimal designs is Bayesian designs and He and Chen (2020) point out that “the Bayesian optimal designs outperform the locally optimal designs when the initial item parameters are poorly estimated.” They observed further in their online calibration setting that locally optimal designs were better than Bayesian optimal designs when the initial estimation of item parameters was based on at least 30 examinees. In the Swedish national tests in Mathematics calibration, the item parameters were based on results of a pretesting from around 230 pupils. Hence, it seems justified to rely on locally optimal designs in our case. Nevertheless, a critical reflection about the quality of the initial parameter estimates is advisable in a general situation. Bayesian optimal designs can be considered if robustness to poor initial estimates is desired.

One of the important decisions in the planning phase of the calibration test is how many test version should be prepared. We investigated the influence of the number of versions on efficiency in this article. The highest efficiency can of course be attained if we allow that each examinee receives an own version. This might be possible making use of the computer-based administration of the items. However, in some cases it might be not desirable from a practical perspective. First, in some cases, versions might need to be created with some manual effort implying an upper bound for the number of versions. Second, it might be good to check if the combination of items in a version make sense. Third, after conduction the test, it can be good to look at results also separately per version, for this we need a not too low number of examinees per version. We recommend to investigate the efficiencies depending on the number of versions. If there is not much gain from increasing the number, one might be satisfied with a lower number, considering the advantages mentioned before.

More and more of the larger achievement tests are administered at the computer. The response time which the examinee used for each item is often collected as well. This was the case also for both the pretesting and the calibration tests considered in this article. For the use of the item in future operational tests, the information about the item response time is also important alongside with other item characteristics like difficulty. Therefore, an interesting option for future research is to also optimize the precision in the estimate for the response time. A basis for optimizing that is to use statistical models for these timings; see, e.g., van der Linden (2007), De Boeck and Jeon (2019), and Sinharay and van Rijn (2020).

## Supplementary Information

Below is the link to the electronic supplementary material.

Supplementary file 1 (pdf 210 KB)

Supplementary file 2 (zip 1 KB)

Supplementary file 3 (r 9 KB)

Supplementary file 4 (r 33 KB)

## Figures and Tables

**Fig. 8:**
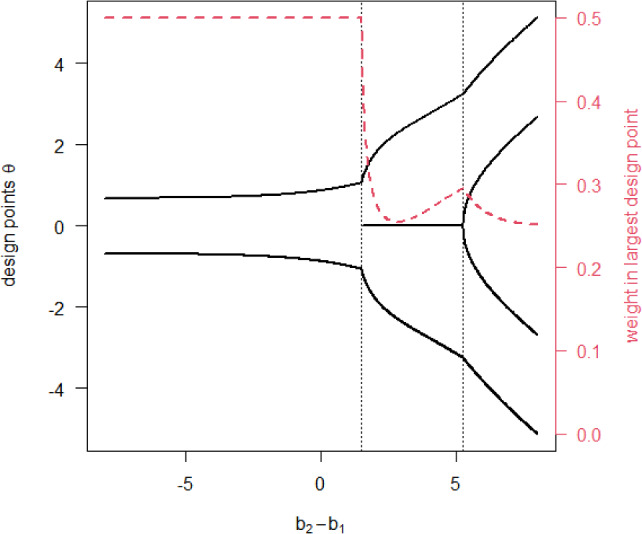
Locally D-optimal unrestricted designs for GPCM with three categories (0, 1, or 2 points) for $a=1$ and $b_1+b_2=0$ depending on $b_2-b_1$. Black solid lines: ability values for design points; red dashed line: weight on largest and smallest design points, where the remaining weight is on the middle or equally divided between the two middle design points; dotted vertical reference lines: $b_2-b_1$-values for change between two-, three-, and four-point designs (approximately at 1.51 and 5.25).

**Fig. 9:**
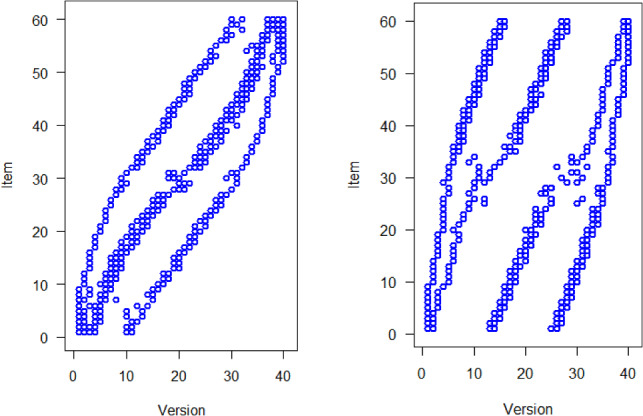
Locally D-optimal designs for GPCM items with $b_{i1}$ equidistantly between -2 and 1, $b_{i2}=b_{i1}+1$ (left panel) or with $b_{i1}$ equidistantly between -2 and 0, $b_{i2}=b_{i1}+2$ (right panel). $a_i=3.5$, 40 versions, 60 items.

**Fig. 10:**
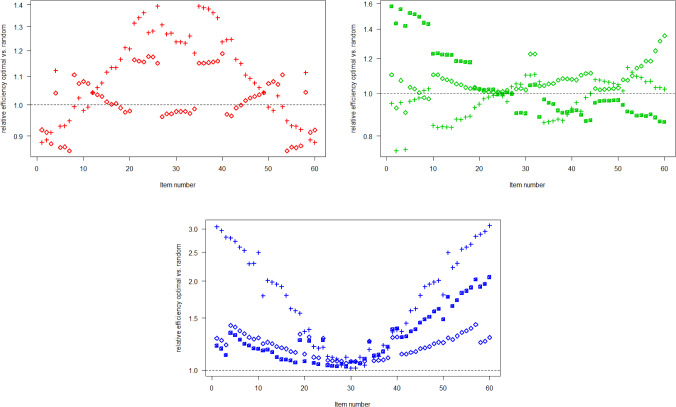
Relative efficiencies for item parameters. First panel 2PL, second panel 3PL, third panel GPCM. Symbols: cross is discrimination parameter *a*, diamond is (first) difficulty parameter *b*, partially filled square is guessing parameter *c* for 3PL or second difficulty parameter for GPCM. 40 versions, and 9 items per version. Optimal versus random design.

## A1: Information Matrix for the GPCM

Following Holman and Berger (2001), we express the GPCM in terms of $f_{ik}(\theta )$ to facilitate different parameterizations

$$\begin{aligned} p_{ik}(\theta ) = \frac{\exp \left\{ f_{ik}(\theta )\right\} }{ \sum _{g=0}^{m_{i}} \exp \left\{ f_{ig}(\theta ) \right\} }, \end{aligned}$$

and the log likelihood terms as

$$\begin{aligned} l_i(\theta )=\sum _{k=0}^{m_i} \log p_{ik}(\theta ) = \sum _{k=0}^{m_i} \left[ \log \{\exp (f_{ik})\} - \log \left\{ \sum _{g=0}^{m_i} \exp (f_{ig})\right\} \right] . \end{aligned}$$

Consider the alternative parameterization of the GPCM with $f_{ik}=a_{i}\cdot k \cdot \theta +c_{ik}.$ For this version of the model there are the following four types of partial derivatives and corresponding elements of the information matrix

$$\begin{aligned} I_{a_i^2} = -E\left( \frac{\partial ^2 l_i(\theta )}{\partial a_{i}^2} \right)&= m_i\theta ^2 \left[ \sum _{g=1}^{m_i}g^2\cdot p_{ig}(\theta ) - \left\{ \sum _{g=1}^{m_i} g\cdot p_{ig}(\theta )\right\} ^2 \right] ,\\ I_{a_i,c_{ik}} = -E\left( \frac{\partial ^2 l_i(\theta )}{\partial a_{i}c_{ik}} \right)&= m_i\theta \left[ k\cdot p_{ik}(\theta )-p_{ik}(\theta ) \left\{ \sum _{g=1}^{m_i} g\cdot p_{ig}(\theta )\right\} \right] , \\ I_{c_{ik}^2} = -E\left( \frac{\partial ^2 l_i(\theta )}{\partial c_{ik}^2} \right)&= m_ip_{ik}(\theta )\left\{ 1-p_{ik}(\theta ) \right\} , \\ I_{c_{ik}, c_{ih}} = -E\left( \frac{\partial ^2 l_i(\theta )}{\partial c_{ik}c_{ih}} \right)&= -m_ip_{ik}(\theta )p_{ih}(\theta ). \end{aligned}$$

### Example: 2-Point Item

For a 2-point item with three response categories we have $k \in \{0, 1, 2\}$, discrimination parameter $a_i$ and location parameters $\textbf{c}_i = (c_{i0}, c_{i1}, c_{i2})$. Setting $c_{i0}=0$ for identifiability leaves $p=3$ parameters to estimate. The information matrix is then given by

$$\begin{aligned} M_i = \int _\Theta \begin{bmatrix} I_{a_i^2} &{} I_{a_i,c_{i1}} &{} I_{a_i,c_{i2}} \\ I_{a_i,c_{i1}} &{} I_{c_{i1}^2} &{} I_{c_{i1},c_{i2}} \\ I_{a_i,c_{i2}} &{} I_{c_{i1},c_{i2}} &{} I_{c_{i2}^2} \end{bmatrix}h_i(\theta ) {\textrm{d}}\theta \end{aligned}$$

with

$$\begin{aligned} I_{a_i^2}&= 2\theta ^2 \left[ p_{i1}(\theta ) + 4p_{i2}(\theta ) - \left\{ p_{i1}(\theta )+2p_{i2}(\theta ) \right\} ^2 \right] , \\ I_{a_i,c_{i1}}&= 2\theta \left[ p_{i1}(\theta ) - p_{i1}(\theta ) \left\{ p_{i1}(\theta )+2p_{i2}(\theta ) \right\} \right] , \\ I_{a_i,c_{i2}}&= 2\theta \left[ 2p_{i2}(\theta ) - p_{i2}(\theta ) \left\{ p_{i1}(\theta )+2p_{i2}(\theta ) \right\} \right] , \\ I_{c_{i1}^2}&= 2p_{i1}(\theta )\{1-p_{i1}(\theta )\}, \\ I_{c_{i2}^2}&= 2p_{i2}(\theta )\{1-p_{i2}(\theta )\}, \\ I_{c_{i1},c_{i2}}&= -2p_{i1}(\theta )p_{i2}(\theta ). \end{aligned}$$

Since D-optimality is invariant with respect to non-singular reparametrisations (Dette & O’Brien, 1999), we can use the information matrix shown above as basis for optimisation after having transformed the parameters $b_{i1}, b_{i2}$ from the pretesting in 2021 to this parametrisation via $c_{i1}=-a_i b_{i1}, c_{i2}=-a_i (b_{i1} + b_{i2})$.

## A2: Locally D-Optimal Designs for GPCM with Three Categories

For the numerical optimization in the case $a=1, b_1+b_2=0$ and a fixed $b_2-b_1$, we considered a symmetrical four-point design

$$\begin{aligned} \xi = \left( \begin{array}{cccc} -x_2 &{} -x_1 &{} x_1 &{} x_2 \\ w &{} 1/2-w &{} 1/2-w &{} w \end{array} \right) , \end{aligned}$$

with $0 \le x_1 \le x_2$ and $0 \le w \le 1/2$. Three-dimensional optimization over $(x_1, x_2, w)$ was done using the BFGS quasi-Newton algorithm with box-restricted boundaries. When one or two parameters where optimal with a value at the boundary ($w=0, w=1/2, x_1=0$, or $x_1=x_2$), it corresponds to a three- or two-point design. The optimal values for $x_1, x_2,$ and *w* are shown in Fig. 8. The optimality of the resulting design was verified with an equivalence theorem by checking the maximum of the directional derivative, see Atkinsson et al. (2007) or Ul Hassan and Miller (2019).

For a general parameter *a*, a scaling argument says that we obtain the same picture, if we have $a(b_2-b_1)$ on the x-axis and divide the values on the y-axis by *a*.

## A3: GPCM Items with Parameters Such That the Optimal Design has More Than Two Regions of Support

We consider two simplified situations with items having GPCM type. We assume again $n=60$ items and $V=40$ test versions, and standard normal distributed abilities. The ability limits $\theta _i$ are the same as in (6). A fixed length restriction (1) is required allowing for $d=9$ items per version.

In both situations, all items follow a GPCM for categories 0, 1, and 2 with discrimination parameter $a_i=3.5$.

- The first situation assumes $b_{i1}$ equidistantly between – 2 (Item 1) and 1 (Item 60) and parameter $b_{i2}=b_{i1}+1$. Hence, $a_i(b_{i2}-b_{i1})=3.5$ for all *i*.
- The second situation assumes $b_{i1}$ equidistantly between – 2 (Item 1) and 0 (Item 60) and parameter $b_{i2}=b_{i1}+2$. Hence, $a_i(b_{i2}-b_{i1})=7$ for all *i*.

Fig. 9 shows the computed optimal designs for the two situations, respectively.

Especially the second situation is of rather theoretical interest only, since it might be hard to create items with both a sharp discrimination of 3.5 between the responses and ICC-cutpoints with two units difference between $b_{i2}$ and $b_{i1}$.

## A4: Relative Efficiencies for Item Parameters

In Fig. 10, we show the efficiencies of the individual item parameters for the three situations of D-optimal designs in Sect. 3.2 (shown in Fig. 2). While the optimal design improves precision of all item parameters in the GPCM, certain parameters in the 2PL and 3PL models show a decrease in efficiency.

It is important to note that the optimization of the D-criterion considers the interplay between parameters (since it optimizes the determinant of the information matrix) and does not singularly optimize two or three parameter estimates independently. The D-criterion has favorable characteristics, including scale invariance and optimality for predicting the item characteristic curve. However, if our primary concern lies in the specific performance of the item parameter estimates, choosing a more tailored criterion, such as A-optimality, might be more suitable.

## A5: Calibration Test for Swedish National Test in Mathematics: Item Characteristics

The item parameters for the 85 items used in the calibration were estimated in a pretest one year prior based on 189 to 269 pupils each (average 229 pupils). Table 5 gives the item parameter estimates and their standard errors from the pretesting. Item 65 and 66 had estimated *a* larger than 5 (8.660 and 12.288, respectively), which was judged unreasonable high and the maximum likelihood estimation was used with an upper bound of 5. Item 13, 24, and 28 were slightly modified which made them easier after pretesting and their difficulty parameters were therefore reduced with 0.3 units. No examinee hat 1 point for the GPCM-Item 41 and the cut-point of the item curves for 0 and 2 points was estimated to be at 0.773. Since a probability of 3-5% for getting 1 point was judged to be reasonable, a restriction of $b_2-b_1\ge -2$ was introduced.

**Table 5:** The items for the calibration test for Swedish national test in mathematics: item parameter estimates and their standard errors, and anticipated response times (in minutes) based on pretesting study. Item types are 2PL, 3PL, or GPCM with 0, 1, or 2 possible points (GP2).

Item	gr^1^	item name	type	*a*	$se_a$	$b_1$	$se_{b_1}$	$b_2$	$se_{b_2}$	*c*	$se_c$	time
1	1	sust_001	2PL	1.348	0.263	0.454	0.151					2
2	2	sust_003	2PL	1.501	0.306	0.983	0.182					3
3	3	sust_005	GP2	0.889	0.178	1.415	0.491	– 1.636	0.52			5
4	4	sust_006	2PL	2.178	0.402	0.332	0.113					5
5	5	sust_007	3PL	0.744	0.205	1.289	0.368			0.001	0.007	3
6	6	sust_022	GP2	0.829	0.156	0.269	0.273	– 0.196	0.272			3
7	7	sust_002a	2PL	1.444	0.294	– 0.905	0.183					5
8		sust_002b	2PL	2.097	0.38	– 0.072	0.112					
9	8	sust_024	2PL	0.822	0.203	0.72	0.244					5
10	9	anny_001a	2PL	1.511	0.254	– 0.232	0.123					3
11		anny_001b	2PL	1.375	0.281	1.245	0.21					
12	10	anny_003	2PL	1.708	0.281	– 0.128	0.113					3
13	11	anny_007	GP2	1.047	0.169	– 0.335	0.226	– 1.143	0.254			5
14	12	anny_008	GP2	1.312	0.224	– 0.411	0.21	– 1.084	0.248			2
15	13	anny_010	2PL	1.478	0.263	0.557	0.134					3
16	14	anny_009	GP2	1.708	0.397	1.705	0.331	0.851	0.24			3
17	15	anny_006a	2PL	1.822	0.378	– 1.632	0.22					3
18		anny_006b	2PL	2.046	0.346	– 0.686	0.124					
19		anny_006c	2PL	2.009	0.345	0.359	0.108					
20	16	sust_020	GP2	0.911	0.15	2.236	0.514	– 1.967	0.508			8
21	17	sust_018	GP2	1.226	0.189	– 0.088	0.147	0.443	0.149			5
22	18	sust_014a	2PL	0.919	0.216	– 2.092	0.439					3
23		sust_014b	2PL	0.982	0.206	1.129	0.231					
24	19	anny_035	GP2	1.505	0.282	0.704	0.257	– 0.21	0.221			8
25	20	anny_036	2PL	1.695	0.302	0.079	0.115					3

**Table 6:** continued

Item	gr^1^	Item name	Type	*a*	$se_a$	$b_1$	$se_{b_1}$	$b_2$	$se_{b_2}$	*c*	$se_c$	time
26	21	anny_038	GP2	0.994	0.176	0.011	0.222	– 0.407	0.231			2
27	22	anny_039	2PL	1.938	0.408	– 0.895	0.144					2
28	23	anny_040	GP2	1.496	0.273	0.023	0.184	– 0.191	0.176			5
29	24	anny_034a	2PL	0.224	0.157	3.047	2.181					2
30	25	anny_025	GP2	0.965	0.174	0.548	0.331	– 1.466	0.379			2
31	26	anny_028	2PL	1.024	0.211	– 0.417	0.171					5
32	27	anny_030a	2PL	1.684	0.325	– 0.905	0.163					3
33		anny_030b	2PL	1.593	0.296	– 0.454	0.135					
34	28	anny_031	GP2	1.211	0.228	1.112	0.27	0.465	0.225			3
35	29	anny_032	2PL	1.752	0.32	0.411	0.123					5
36	30	anny_026a	2PL	0.682	0.215	– 2.431	0.703					3
37		anny_026b	2PL	0.928	0.2	0.205	0.172					
38	31	anny_029a	2PL	3.234	0.709	– 0.233	0.096					3
39		anny_029b	2PL	2.756	0.556	– 0.072	0.098					
40	32	join_003	GP2	0.924	0.176	2.827	0.583	– 0.205	0.421			8
41	33	join_004	GP2	1.982	0.353	1.773	0.129	– 0.227	0.129			5
42	34	join_005	2PL	1.078	0.228	– 0.998	0.214					3
43	35	join_007	2PL	2.033	0.424	1.556	0.202					5
44	36	join_008a	2PL	1.793	0.313	– 0.036	0.109					5
45		join_008b	2PL	2.629	0.484	0.039	0.094					
46		join_008c	2PL	2.981	0.582	0.468	0.098					
47	37	join_009a	2PL	1.561	0.645	– 3.134	0.752					3
48		join_009b	2PL	1.454	0.214	– 1.656	0.992					
49		join_009c	2PL	1.161	0.203	– 0.692	0.234					
50	38	join_011	2PL	2.143	0.238	– 0.083	0.146					3

**Table 7:** continued

Item	gr^1^	Item name	Type	*a*	$se_a$	$b_1$	$se_{b_1}$	$b_2$	$se_{b_2}$	*c*	$se_c$	time
51	39	join_012	3PL	2.541	0.217	– 0.775	0.478			0.274	0.044	3
52	40	join_013	2PL	1.709	0.271	– 1.521	0.338					2
53	41	join_014	2PL	3.883	0.593	– 1.302	0.193					2
54	42	join_016	2PL	0.748	0.181	1.126	0.58					5
55	43	anny_044	3PL	1.883	1.458	1.035	0.262			0.246	0.108	2
56	44	anny_043	2PL	1.086	0.248	– 1.465	0.292					2
57	45	anny_045a	2PL	2.229	0.403	0.082	0.106					3
58		anny_045b	2PL	2.014	0.361	0.25	0.113					
59	46	anny_048	2PL	1.077	0.218	– 0.5	0.17					2
60	47	anny_046a	2PL	1.48	0.376	– 1.877	0.33					3
61		anny_046b	2PL	2.931	0.599	– 0.438	0.102					
62		anny_046c	2PL	2.289	0.426	– 0.325	0.107					
63	48	anny_049a	2PL	0.771	0.185	0.723	0.247					5
64		anny_049b	2PL	1.006	0.205	0.057	0.162					
65	49	sust_010a	2PL	5	3.35	– 0.162	0.076					5
66		sust_010b	2PL	5	7.906	– 0.128	0.075					
67		sust_010c	2PL	3.417	0.653	0.372	0.092					
68	50	join_031	3PL	0.753	0.191	0.721	0.259			0.001	0.03	2
69	51	anny_075	3PL	1.925	0.366	– 0.28	0.131			0.001	0.04	2
70	52	anny_056	2PL	1.307	0.251	0.59	0.154					3

**Table 8:** continued

Item	gr^1^	Item name	Type	*a*	$se_a$	$b_1$	$se_{b_1}$	$b_2$	$se_{b_2}$	*c*	$se_c$	time
71	53	anny_069a	2PL	1.305	0.292	– 1.642	0.285					2
72		anny_069b	2PL	1.362	0.261	– 0.901	0.175					
73	54	anny_084a	2PL	1.595	0.397	– 1.951	0.319					3
74		anny_084b	2PL	1.482	0.309	– 1.341	0.218					
75	55	anny_051	3PL	2.337	1.392	0.807	0.232			0.292	0.073	2
76	56	anny_058	3PL	4.972	7.696	0.657	0.224			0.426	0.074	2
77	57	anny_077	2PL	1.482	0.35	1.092	0.211					3
78	58	anny_068	GP2	0.675	0.142	2.023	0.706	– 2.625	0.766			5
79	59	anny_082	2PL	1.121	0.249	0.412	0.178					3
80	60	anny_079	3PL	0.147	7.156	14.163	1.4			0.001	0.027	5
81	61	anny_063	GP2	0.942	0.177	1.168	0.4	– 1.184	0.408			5
82	62	anny_083	3PL	1.005	0.255	– 1.61	0.369			0.003	0.017	3
83	63	anny_062a	2PL	1.472	0.324	– 1.416	0.247					5
84		anny_062b	2PL	0.902	0.265	– 2.298	0.579					
85		anny_062c	2PL	0.662	0.219	– 2.291	0.708					

gr = item group number
