# Supplementary material for: Parallel Optimal Calibration of Mixed-Format Items for Achievement Tests
Source: Psychometrika. 2024 Apr 15;89(3):903–28. doi: 10.1007/s11336-024-09968-3 (PMC11458722; doi:10.1007/s11336-024-09968-3)
Supplement: Supplementary file 1 — (pdf 210 KB) [file 11336_2024_9968_MOESM1_ESM.pdf]

Psychometrika
Supplementary Materials for Section 3 of "Parallel Optimal Calibration of Mixed-Format Items for Achievement Tests"

Frank Miller[1,2] and Ellinor Fackle-Fornius[1]

[1]Department of Statistics, Stockholm University,

[2]Department of Computer and Information Science, Linköping University

Correspondence should be sent to
E-Mail: frank.miller@liu.se

S1 More Results for the 2PL Model

In Section 3.2, we showed results for $V = 40$ versions. For the 3PL and GPCM, we have seen that relative item efficiencies (optimal versus random design) tended to be higher for easy and difficult items. For the 2PL model, this was not as clear. However, when looking at other number of versions (Figure 1 for $V = 20, 60$), we see this pattern also for the 2PL model.

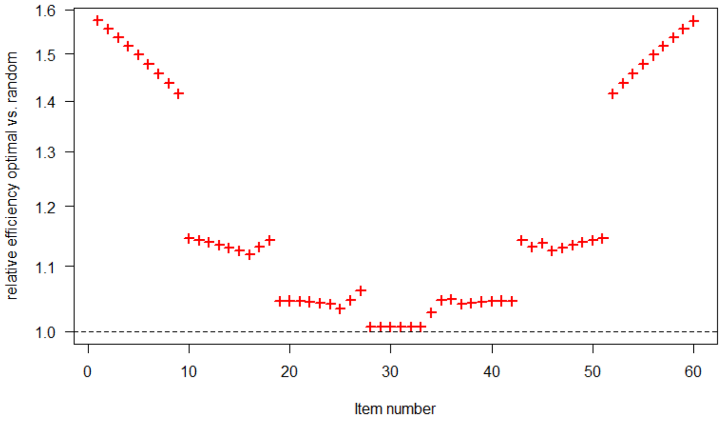

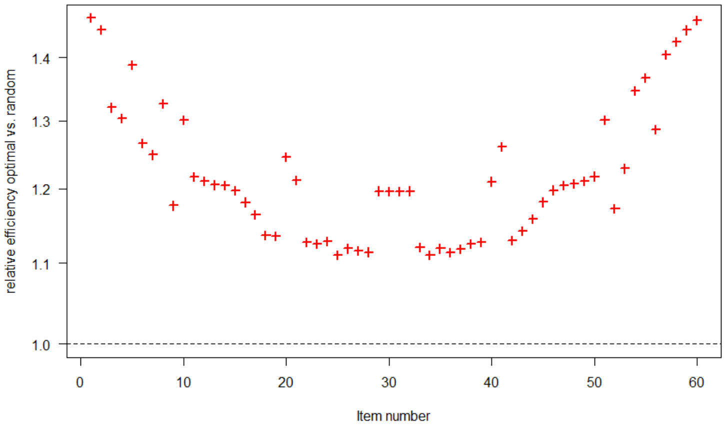

Figure 1: Relative efficiencies for 2PL model with 20 versions (first panel), and 60 versions (second panel), and 9 items per version. Optimal vs. random design.

S2 Influence of Item Response Times on Relative Efficiencies

As described in Section 2.4, we optimized the determinant of the total information matrix, or equivalently the product of the item-information matrices' determinants. In the case study, each version was restricted to have 40 minutes expected time for the test. Items with lower item-response-time are preferred by the optimization since more of them can be used in a version and they then deliver more total information. On the other hand, the product criterion ensures that each item is used sufficiently, including items with longer item-response-time.

Table 1 shows the average number of versions an item is used in. It is clear that increasing item-response-time implies use in a decreasing number of versions. 2PL items with 5 minutes item-response-time and GPCM items with 8 minutes item-response-time are used in 2 versions, only. Item groups of three items with 3 minutes item-response-time are used in average in 7.7 versions since they give information about 6 model parameters in a relatively short time.

Item type	# model parameters	resp. time (min.) 2	3	5	8
2PL (single item)	2	3.4	2.8	2.0	
3PL	3	5.4	3.3	3.0	
GPCM	3	6.7	4.0	2.6	2.0
2PL (two-item-group)	4	7.0	5.3	3.5	
2PL (three-item-group)	6		7.7	5.0	

Table 1: Calibration test for Swedish national test in Mathematics: Average number of versions an item is used in depending on item-response-time and item type.

When comparing optimal with random design, the relative efficiency for an item depends on how many pupils are allocated to this item (i.e. how many versions uses this item). Figure 2 shows this dependency.

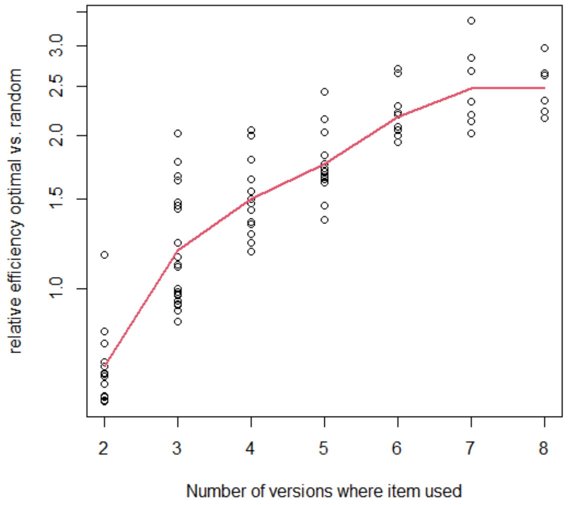

Figure 2: Calibration test for Swedish national test in Mathematics: Relative efficiencies depending on number of versions the item is used in (black dots). Geometric mean efficiencies for each number of versions (red line).

S3 Bias of Parameter Estimates After Optimal and Random Design: A Simulation Study

We consider the 2PL and 3PL scenarios from Section 3.2 with 60 pretest items. We assume that $N = 1600$ examinees participate in a calibration test and that their abilities were estimated in a larger operational test, in such a way that each ability parameter was estimated approximately unbiased, with standard deviation 0.25.

For the simulation study, we generate the true abilities of all examinees (which are unknown in reality) from a standard normal distribution. The known estimates of the abilities are generated by adding a $N(0, 0.25^2)$-distributed random variable to the true ability. Next, we generate answers to the pretest items based on the true abilities, the chosen model (2PL or 3PL), and the design (D-optimal or random). In both designs, each examinee was allocated to 9 pretest items and both designs used 40 versions, where the items were randomly placed for the random design.

The item parameters were then estimated by maximum likelihood estimation based on the estimated ability (true ability + $N(0, 0.25^2)$). We

conducted 1000 repetitions.

Figure 3 shows the bias for the two parameters in the 2PL model for the D-optimal and the random design (mean over the 1000 replications). Figure 4 shows bias for the three parameters in the 3PL model for the D-optimal and the random design (median over the 1000 replications). For the 3PL model, the algorithm for maximum likelihood estimation did not always converge in the 60000 cases (60 items times 1000 repetitions); for the D-optimal design, we had 4 non-convergences out of 60000; for the random design, we had 74 non-convergences. When a non-convergence occurred for one design, this simulation run was excluded from the summary plots for both designs. Further, a small number of very extreme parameter estimates for discrimination and ability were obtained; therefore, we show the medians instead of the means over the 1000 repetitions.

We see that there are biases (mean or median) for both designs. For these scenarios we see that the biases for both models as well as non-convergence issues for the 3PL are clearly reduced with the D-optimal design compared to the random design. The reason is that the D-optimal design includes the right person information to avoid that too little information is obtained for estimating the parameters.

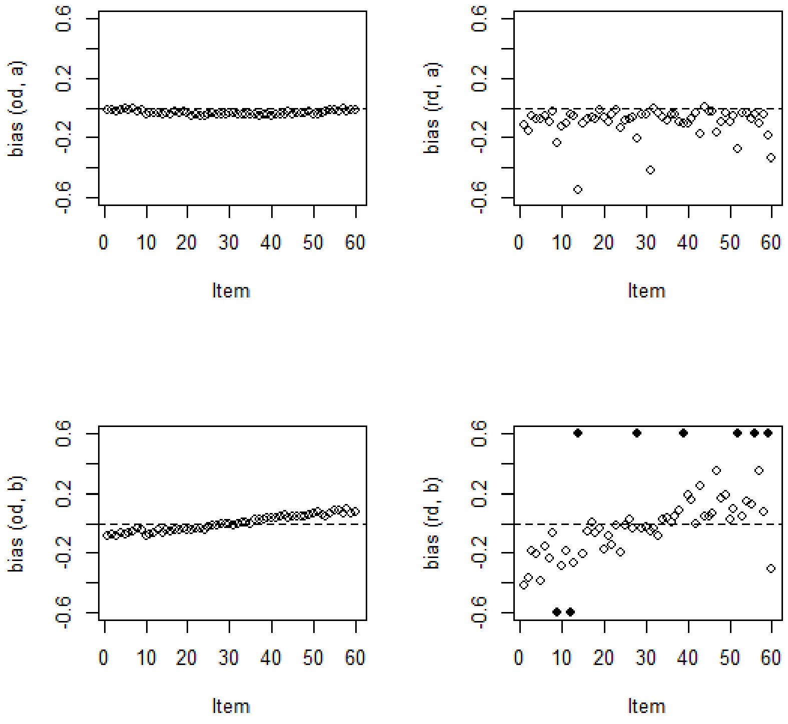

Figure 3: 2PL model: Bias of parameter estimates after D-optimal (od, left panels) and random (rd, right panels) design; upper panels for discrimination parameter a, lower panels for difficulty parameter b. Mean over 1000 repetitions; filled dots indicate higher or lower values outside the plotting region.

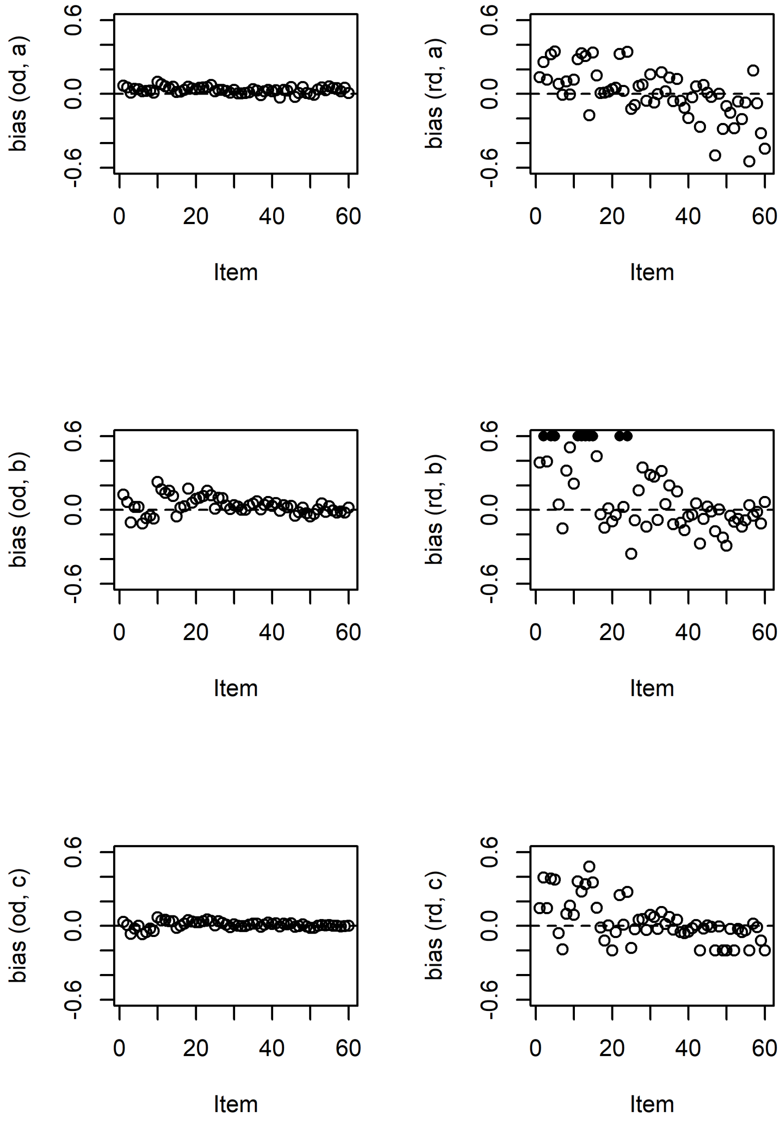

Figure 4: 3PL model: Bias of parameter estimates after D-optimal (od, left panels) and random (rd, right panels) design; upper panels for discrimination parameter a, middle panels for difficulty parameter b, lower panels for guessing parameter c. Median over 1000 repetitions; filled dots indicate higher or lower values outside the plotting region.
